# Fine-Scale Spatial Variability of Pedestrian-Level Particulate Matters in Compact Urban Commercial Districts in Hong Kong

**DOI:** 10.3390/ijerph14091008

**Published:** 2017-09-03

**Authors:** Yuan Shi, Edward Ng

**Affiliations:** 1 School of Architecture, The Chinese University of Hong Kong, Shatin, NT, Hong Kong, China; edwardng@cuhk.edu.hk; 2 Institute of Environment, Energy and Sustainability (IEES), The Chinese University of Hong Kong, Shatin, NT, Hong Kong, China; 3 Institute of Future Cities (IOFC), The Chinese University of Hong Kong, Shatin, NT, Hong Kong, China

**Keywords:** particulate matters, fine-scale spatial variability, pedestrian level, geospatial interpolation

## Abstract

Particulate matters (PM) at the pedestrian level significantly raises the health impacts in the compact urban environment of Hong Kong. A detailed investigation of the fine-scale spatial variation of pedestrian-level PM is necessary to assess the health risk to pedestrians in the outdoor environment. However, the collection of PM data is difficult in the compact urban environment of Hong Kong due to the limited amount of roadside monitoring stations and the complicated urban context. In this study, we measured the fine-scale spatial variability of the PM in three of the most representative commercial districts of Hong Kong using a backpack outdoor environmental measuring unit. Based on the measurement data, 13 types of geospatial interpolation methods were examined for the spatial mapping of PM_2.5_ and PM_10_ with a group of building geometrical covariates. Geostatistical modelling was adopted as the basis of spatial interpolation of the PM. The results show that the original cokriging with the exponential kernel function provides the best performance in the PM mapping. Using the fine-scale building geometrical features as covariates slightly improves the interpolation performance. The study results also imply that the fine-scale, localized pollution emission sources heavily influence pedestrian exposure to PM.

## 1. Introduction

Over the past few decades, the adverse impacts of urban air pollution on public health have been increasingly identified as a global problem [1,2]. Pedestrians in the urban outdoor space are more and more often exposed to harmful ambient environments with different air pollution sources (including but not limited to the traffic-related pollution, household air pollution, and commercial cooking smoke exhaust from roadside buildings) [3]. The human exposure to the particulate matters (PM), for example, PM_2.5_ (particles with an aerodynamic diameter <2.5 μm) and PM_10_ (particles with an aerodynamic diameter <10 μm), has also been associated with many negative health outcomes [4,5]. In a highly urbanized area, the dense building clusters form street canyons with heavy motor-traffic flows. Under such circumstance, the air movements are stagnant [6] and the pollutant dispersion is significantly decelerated [7]. As a consequence, the health risks of pollution exposure in such pollutant-concentrated street environment will be considerably increased [8]. 

Hong Kong, as one of the most densely populated cities around the world, has an extremely compact urban environment [9]. Pedestrians are exposed to severe air pollution from the motor traffic [10,11,12]. PM exposure has been investigated and proved to be strongly associated with health burdens [13,14]. The intensive urban development also makes the distribution of pollution emission sources (both traffic and fixed point source emissions) more complicated and the relevant analysis has to be performed at a much finer scale. Such context leads to a considerably high spatial variability of the particulate matters at a fine scale (a microenvironmental scale). It has been emphasized that monitoring the spatial changes of urban air quality is essential [15]. The above indicates the need for a detailed investigation of the fine-scale spatial variation of air quality in the compact and diverse urban environment of Hong Kong in the assessment of health risk to pedestrians in the outdoor environment.

However, the PM data monitored by the local air quality monitoring network managed by the government authorities cannot provide microenvironmental scale information for individual-level health risk assessments [16]. In Hong Kong, the hourly air quality condition is currently monitored by a local air pollution monitoring network managed by the Environmental Protection Department of Hong Kong (HKEPD). Among the 15 stations of this network, only three are placed on the roadside [17]. The real challenge in the investigation of the spatial variability of air quality is that Hong Kong has an extremely heterogeneous built environment. This heterogeneity results in large variations between different locations of the city. Even in a single district, conditions cannot be effectively observed by the three fixed roadside air quality stations. There will be large uncertainties and errors in using the PM data of a fixed station for the pedestrian health risk assessment [15]. Many efforts have been made to investigate the pedestrian exposure to PM in urban sites of Hong Kong with heavy traffic conditions [18,19,20,21], but they are all based on the data from a couple of fixed monitoring locations. The neural network is also a useful method of forecasting the traffic-related pollutant concentrations [22]. In Hong Kong, another attempt of forecasting the air quality in a dense commercial district has been made by developing a neural network model [23]. However, it only provides temporal forecast but not spatial estimation. The lack of the information of fine-scale spatial variability is still a major limitation in the evaluation of individual exposure to the pedestrian-level air pollution [24,25].

To overcome the above limitations, this study aims to initially investigate the fine-scale spatial variability of PM in the typical compact urban environment by conducting a pilot test of pedestrian level PM measurement by walking through selected urban areas. The geostatistical technique will be used to analyse the data and to map the microenvironmental PM spatial variation. The microenvironmental scale mapping of pedestrian-level PM provides better spatial information for exposure assessment and environmental management. The experience from this initial study and pilot test will provide a valuable knowledge basis for further environmental mapping studies and individual exposure assessments in Hong Kong. 

## 2. Materials and Methods 

In this study, a pilot test was designed and performed. First, we measured the fine-scale spatial variability of the PM_2.5_ and PM_10_ in three of the most representative compact commercial districts of Hong Kong using a self-assembled backpack outdoor environmental measuring unit (temperature, humidity, PM concentration, and real-time geographical locations). Based on the spatial PM onsite measurement data, geostatistical semivariogram modelling was then performed and used as the basis of spatial interpolation of the PM data. A total of 13 types of geospatial interpolation methods were examined for the spatial mapping of PM_2.5_ and PM_10_ with a group of building geometrical/urban setting covariates. The optimal method was determined by comparing the interpolation performance. Finally, geospatial mapping of PM_2.5_ and PM_10_ was conducted by using the optimal interpolation method determined by the above step. Cross validation was performed to examine the prediction performance of the resultant geospatial mapping.

### 2.1. Study Areas

**Figure 1:**
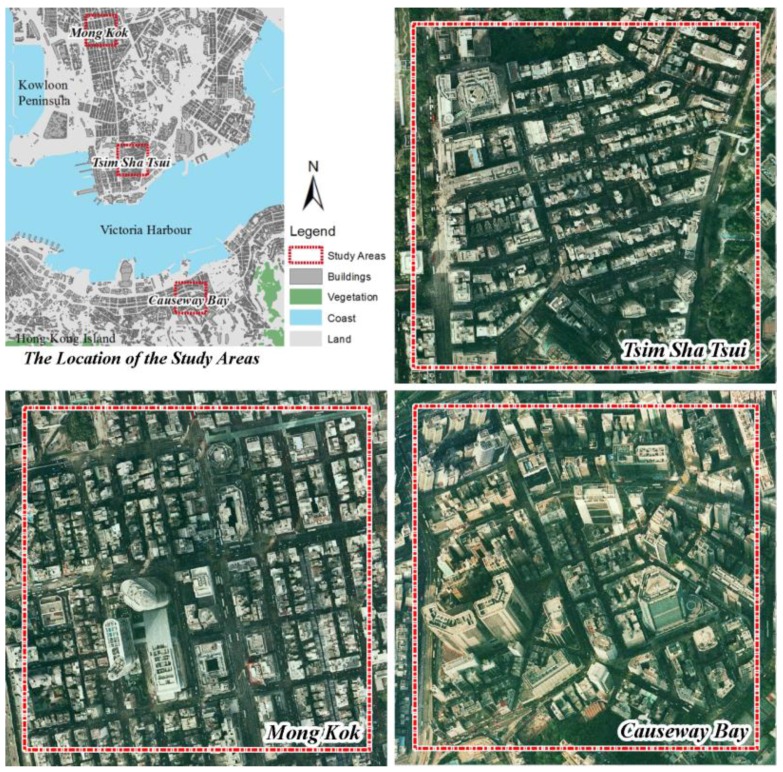
The location and the building morphology of the three study areas (the size of the red rectangle is 500 × 500 m).

Three study areas—Mong Kok, Tsim 56Sha Tsui and Causeway Bay—were selected for this pilot study of microenvironmental PM mapping (Figure 1). Mong Kok and Causeway Bay are two generally concerned hotspot districts of pedestrian level air pollution where the HKEPD roadside stations are placed [26]. Tsim Sha Tsui is also one of the best known commercial districts and tourist sightseeing areas located in the compact downtown area of the Kowloon peninsula. However, it is not monitored by any HKEPD roadside station despite being a dense urban core with heavy traffic. Therefore, it is also selected as a study area.

With regards to the population density, all three districts are extremely densely populated. They all have a population density over 30,000 people/km^2^ (the average population density of Hong Kong is about 7000 people/km^2^). As the best known and representative compact commercial districts of Hong Kong, these three districts are quite similar in terms of land use, urban functions (highly mixed high-rise commercial mansions, shopping centers with some densely built residential buildings), and traffic conditions (intensive traffic and pedestrians’ outdoor activities). The activities and behaviors of the pedestrians in these three districts are also similar (leisure and entertainment, shopping). Moreover, the types of the fine-scale emission sources in these three districts are also similar (as mentioned in the paper, vehicular pollutants, the densely distributed bus stops [27], restaurants and commercial cooking [28], etc.). The above similarities not only provide a representative context for investigating fine-scale human exposure but also make the three an ideal study site group for observing the effect of buildings on the fine-scale pollution dispersion.

These three intraurban areas represent two distinct urban morphological characteristics. As one of the most famous urban commercial districts and tourist attractions in Hong Kong, Mong Kok has a road network and a zoning plan based on a classical orthogonal grid. This orthogonal grid layout has been widely used in urban planning in intraurban areas with flat terrain (Many famous large cities around the world have the similar orthogonal grid layout such as Manhattan in New York, Vancouver and Barcelona [29]). Compared with Mong Kok, both Tsim Sha Tsui and Causeway Bay have a more irregular urban road network due to the irregular terrain and the costal location. Mong Kok also has a relatively lower and more homogeneous building height distribution than Causeway Bay. The building morphology of the Tsim Sha Tsui area is more similar to the Causeway Bay, but with a more compact layout.

The urbanization process significantly changes the aerodynamic roughness in the urban area [30,31]. It consequently alters the near-surface wind field and weakens the dispersion of air pollution [32]. The compact urban morphology in Hong Kong, especially in the three study areas, shapes the very deep street canyons (implying a slower dispersion) with large traffic volume (leading to a larger emission intensity). Therefore, it would be helpful to incorporate the consideration of urban morphology into the geospatial analysis of this study. 

### 2.2. Field Measurement

**Figure 2:**
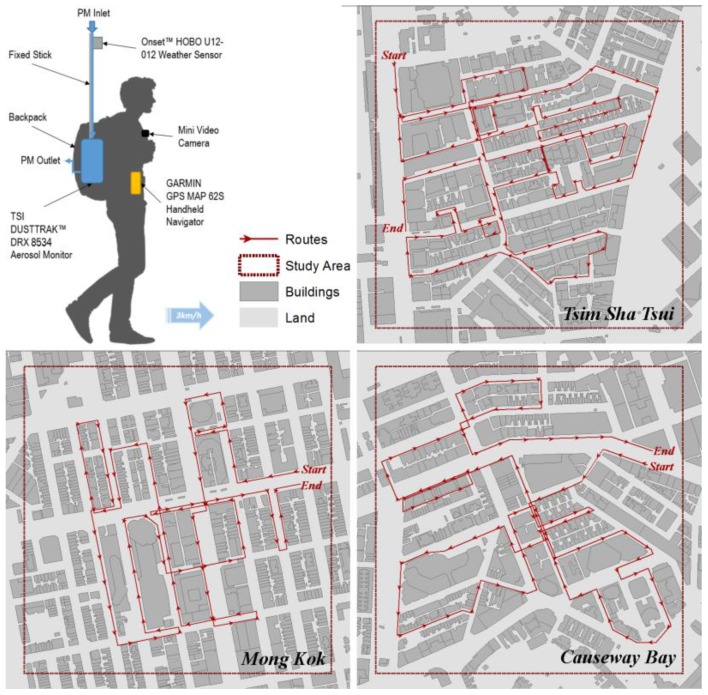
The instrumentation of the backpack measuring unit, and the walking measurement routes in the three selected study areas for measuring the pedestrian-level PM concentration. (The walking measurement routes shown in this figure are labelled based on the forward direction of the walkthrough).

A self-assembled backpack outdoor environmental measuring unit was prepared for the field measurement (Figure 2). The PM_2.5_ and PM_10_ were continuously sampled using the TSI DUSTTRAK™ DRX Aerosol Monitor Model 8534 (the DUSTTRAK monitor) with a sampling interval of 1 s. The inlet sampling tube was installed at the height of 2.00 m above the ground surface in order to reflect the pedestrian-level condition without random influence by near temporary emission sources that are out of the study scope (e.g., smokers). Before the field measurement, the advanced calibration procedure of the DUSTTRAK monitor (recommended by the manufacturer) was conducted based on the concentration data from the simultaneous gravimetric PM_2.5_ and PM_10_ sampling at a local air quality monitoring station [33] (the Mong Kok roadside station). Air temperature (*T_a_*, °C) and relative humidity (*RH*, %) were simultaneously measured by an Onset™ HOBO U12-012 weather sensor (HOBO). The HOBO-measured data were used for the *RH* calibration of the measured PM_2.5_ and PM_10_ data. The DUSTTRAK-measured data were corrected using simultaneously measured relative humidity (*RH*) and the following equation [34]:

(1) $$Correction\;Factor=1+0.25\frac{RH^{2}}{\left(1-RH\right)}$$

In Hong Kong, the pedestrian level wind speed is already very low [35] under a calm or light wind condition. The backpack measurement unit is moving (similar to a typical pedestrian on the streets of Hong Kong) which means that it is difficult to get a precise measurement of wind speed. Global solar radiation is not measured as well, as the compact urban morphology is already a modifying factor of the wind speed and solar flux (the building morphological factors were selected as the covariates for the geointerpolation of this study). A GPS logger (Garmin^TM^ GPS 62 s model, with an accuracy of location within 4.0 m) was used to record the corresponding geographical location of each measurement data. All the instruments were synchronized to Coordinated Universal Time (UTC) to make sure that the data logging timeline is synchronized. 

Due to the seasonal variation of the regional weather system, the air pollution condition in Hong Kong is dominated by the regional air pollution (with a higher PM concentration) during the winter time [36]. This regional-dominant pollution mode affects Hong Kong one-third of the time in a year [37]. In this study, the regional-dominant influence of the long-distance transportation of air pollution from the Pearl River Delta (PRD) region of mainland China is out of the study scope. The air quality is dominated by local emission sources during summer time [20,38]. In order to minimize the influence of high background concentration [20], all measurement campaigns were performed under the typical summer weather condition of Hong Kong (no rainfall; partially cloudy [39]; calm or light wind condition [7,40]—a Beaufort wind scale <2 in this study; *RH* around 85% according to the Hong Kong Observatory [41]). All the three sampling sessions for the three study areas were performed from June to July 2015 (typical summer without extreme weather condition/events). The diurnal pattern of the PM concentration level monitored at the Mong Kok roadside air quality monitoring station of local authority (HKEPD) has been investigated. A considerable increase of PM concentration was observed between 6:00 a.m. and 10:00 a.m. (caused by the surge in traffic of morning commuting). The roadside PM concentration level also has a rapid decline after the evening traffic-intensive hours (in Hong Kong the evening rush hour usually lasts at least until 8:00 p.m. due to the overtime works). Considering above diurnal pattern of roadside PM and also pedestrian activities, all measurement sessions were performed within the time range of 2:00 p.m.–8:00 p.m. (during which the hour-to-hour changing gradient of background PM concentration is smaller than other hours). 

During the field measurement, a measurer keeps walking through the study area along a designated route at a typical pedestrian walking speed of 3 km/h (0.8 m/s) [42] carrying the calibrated instruments, so the spatial variation of PM concentration at pedestrian level can be well observed. There are both a forward and a backward walkthrough along the route in each measurement session to eliminate the bias of the different walking directions along the route. Even in an extremely crowded street environment with fast-paced traffic flow, the measurement was still designed to gather spatial information as comprehensive as possible. During the measurement campaigns, at those streets where the pedestrian crossing is available, the person was also asked to walk through the both sides of the street instead of only sampling the PM_2.5_ concentration level on a single side of the street. The data measured while crossing the street were also kept to understand the PM_2.5_ concentration at the road center. The maximum walking time is set to 2 h for each study area to make sure that the background concentration level and weather conditions have no significant changes. 

### 2.3. Geospatial Interpolation Methods

Spatially continuous data of air pollution concentration play an important role in depicting urban air quality, but acquiring such data is not an easy task [43,44]. Different approaches have been applied to acquire spatially continuous air pollution concentration data, including remote sensing methods [45], computational fluid dynamics (CFD) simulation [46], and geographical mapping [47]. Remote sensing data have been used for mapping the spatial distribution of ground-level PM_2.5_ in Hong Kong but only at a large scale with a coarse spatial resolution [48,49]. CFD methods become more popular in modeling the microenvironment air pollution, but there are still uncertainties due to the limitation of either the turbulent models or the computational resources [50]. The geographical mapping methods (based on real measured data, as a cost-effective way) have been used to map the microclimatic spatial distribution in the high-density urban environment of Hong Kong [51]. The results show a reasonably good mapping accuracy with high practicability when dealing with the complicated built environment of Hong Kong. Therefore, the geographical mapping method was selected as the main method of this study as well. 

Spatial interpolation is the key of geographical mapping. In this study, 13 types of spatial interpolation methods were tested and compared to map the microenvironmental spatial distribution of PM_2.5_ and PM_10_ (Table 1). The effects of building geometrical features measured by the sky view factor (SVF) and frontal area index (FAI) have been identified as determinant factors of the urban scale spatial variability of PM in Hong Kong [16,52]. In these studies, the road area ratio (RDA) as an indicator of the traffic capacity/volume of the spatially arranged road network was also proved to be an important predictor of the spatial variability of PM, because it largely reflects the traffic volume distribution under the extremely compact urban scenario of Hong Kong. In this present study, these three factors were calculated within the range of a microenvironmental scale (defined as a buffer range of 50 m) and considered as weight factors/covariates of the spatial interpolation to test whether they are still the dominants of the air pollution spatial distribution at the microenvironment scale. Implementation of different types of interpolation methods was based on previous studies and several practical guides of geointerpolation [53,54]. The building geometry data were used as the barrier layer in the KIB algorithm.

A spatial interpolation is based on the assumption that the spatial variation in the study area can be explained by a spatial correlation between data points which is a function of distance [55]. The LPI method uses the polynomials to fit complex curves with the measured data points, and uses these spatial curves to create continuous prediction surface of the spatial variation. The kriging method (OK and OCK in this present study) develops the prediction surface by weighting the surrounding measured data points (based on a semivariogram model) to estimate the value of unmeasured locations [56]. Below formula demonstrates how a spatial interpolation works:

(2) $$\hat{Z}\left(S_{unmeasured}\right)=\overset{N}{\underset{i=1}{\sum }}\lambda_{i}Z(S_{i})$$

where $\hat{Z}\left(S_{unmeasured}\right)$ is the interpolated (predicted) value at a unmeasured location. *N* is the total amount of the measured locations. $Z(S_{i})$ is the real measured value at the location *S_i_* in the study area. $\lambda_{i}$ is the weighting factor of the $Z(S_{i})$ resulting from fitted curves/models.

There are two important elements that need to be examined in performing the above interpolations—the kernel functions and the spatial correlation of the data. The polynomials used in the LPI depends on a kernel function (the geointerpolation fits a prediction surface based on a kernel function). In this study, six types of commonly used kernel functions were tested using the concentration value of PM_2.5_ and PM_10_ of each study area to determine the optimal kernel function for the purpose of achieving the minimum interpolation error, evaluated using the root mean square error (RMSE). A *k*-fold cross validation was also adopted to avoid bias. See Section 2.4 of this article). Kernel functions that produce the minimum RMSE should be used for the further mapping process. Some interpolation methods are based on the kernel function (e.g., LPI, KIB) while many other spatial interpolation methods are by the semivariogram modelling such as OK and OCK methods. Geostatistical analysis was used to determine the optimal semivariogram model of PM_2.5_ and PM_10_ of each study area for further spatial interpolation. The semivariogram is measured as follows:

(3) $$\gamma \left(d_{ij}\right)=\frac{1}{2n\left(d_{ij}\right)}\overset{n\left(d_{ij}\right)}{\underset{s_{i}-s_{j}=d_{ij}}{\sum }}{\left[Z(S_{i})-Z(S_{j})\right]}^{2}$$

where $\gamma \left(d_{ij}\right)$ is the semivariogram. There are $n\left(d_{ij}\right)$ pairs of spatial locations of measured data in the study area. $d_{ij}$ is the spatial distance between the location *S_i_* and *S_j_*. $Z(S_{i})$ and $Z(S_{j})$ are the measured value at the location *S_i_* and *S_j_* in the study area. A semivariogram $\gamma \left(d_{ij}\right)$ model is a function of *d*, which depicts the spatial correlation of the value of interest in a certain spatial range. This spatial correlation could provide an estimation for those unmeasured locations between two measured locations. It is the basis of spatial interpolation. It is also an important indicator of the spatial independence of the data. Using the empirical semivariogram modeling method, we not only develop the semivariogram models as the basis of the further interpolation but also test the major range of the spatial independence of PM_2.5_ and PM_10_ [56]. The spatial independence enables the determination of the optimal spatial scale of representing the spatial variability of the data separately for the three study areas. The ArcGIS software (the embedded Geostatistical Analyst module) was used for the above geospatial analysis [56].

### 2.4. The Validation and Comparison of Interpolation Methods

In this study, both the leave-one-out cross validation (LOOCV) and the *k*-fold cross validation were adopted to validate all resultant spatial interpolation results of PM and compare the prediction performance of different interpolation methods. In a LOOCV a predicted dataset is firstly generated. This dataset includes all predicted values from interpolation mapping for each corresponding location of the measured points. Then, a simple linear regression (SLR) between the predicted value dataset and measured value dataset is developed. The RMSE of the SLR (between predicted and measured data) was calculated as follows:

(4) $$RMSE=\sqrt{\frac{1}{n}\overset{n}{\underset{i=1}{\sum }}{\left(PM_{i}^{\prime }-PM_{i}\right)}^{2}}$$

where $PM_{i}$ is the measured value of the PM concentration at the at the point location *i* of the study area. $PM_{i}^{\prime }$ is the estimated PM concentration at the pixel *i* (the corresponding pixel of the location *i*) in the resultant spatial interpolation mapping. To avoid estimation bias near the much localized fine-scale pollution emission sources, a *k*-fold cross validation with *k* = 2 was also performed. In this 2-fold cross validation, the measured PM data was divided into two sets. The first data set is used as the model training dataset, while the other set was used as the evaluation dataset. The above process was performed twice so that each set can be used as both the training dataset and the evaluation dataset. The $R{\;}_{k-fold}^{2}$ was calculated and used for the evaluation [57]. Both the RMSE and the $R{\;}_{k-fold}^{2}$ were also used for the comparison of the kernel functions and the final prediction performance of the 13 types of interpolation methods mentioned above (in Table 1).

## 3. Results

**Figure 3:**
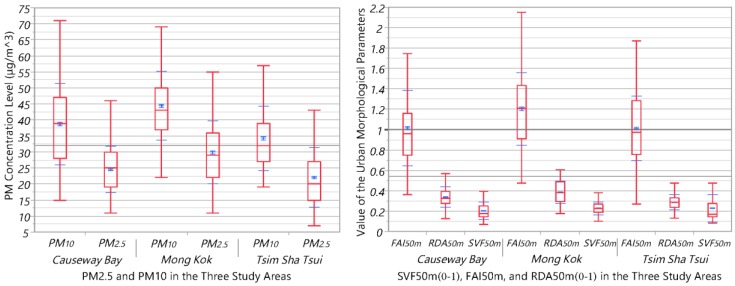
The quantiles box plots (10%, 25%, 50%, 75% and 90%; Mean and Standard Deviation) of PM_2.5_ and PM_10_ concentration levels and the three covariates (SVF_50m_, FAI_50m_ and RDA_50m_) in the three study areas.

As described in the methodology section, the optimal kernel function was identified first. Then, the semivariogram models were developed for each study area to determine the optimal spatial scale of representing the data variation. On top of that, a total of 13 types of methods were to map the fine-scale spatial variability of PM_2.5_ and PM_10_ in the three study areas. During this process, three building geometrical-related weight factors/covariates were examined. Finally, the prediction performance was compared to determine the best interpolation method. Figure 3 shows the general statistics of PM_2.5_ and PM_10_ concentration levels and the three covariates (SVF_50m_, FAI_50m_ and RDA_50m_) in the three study areas.

### 3.1. The Optimal Kernel Functions

The results (Table 2) show the RMSE and the $R{\;}_{k-fold}^{2}$ of using six types of kernel functions for PM_2.5_ and PM_10_ of the three study areas. The comparison results show that the exponential kernel function produces the minimum RMSE and the highest $R{\;}_{k-fold}^{2}$ values of the estimation of the spatial variation in both the PM_2.5_ and PM_10_ concentration in all three study areas. Therefore, the exponential kernel function is used for further interpolation. The equation below shows an exponential kernel function for geointerpolation (where r is the radius of a center point, h is the bandwidth) [56].

(5) $$K\left(\frac{r}{h}\right)=e^{-3\left(\frac{r}{h}\right)}$$

### 3.2. The Semivariogram Modelling

**Figure 4:**
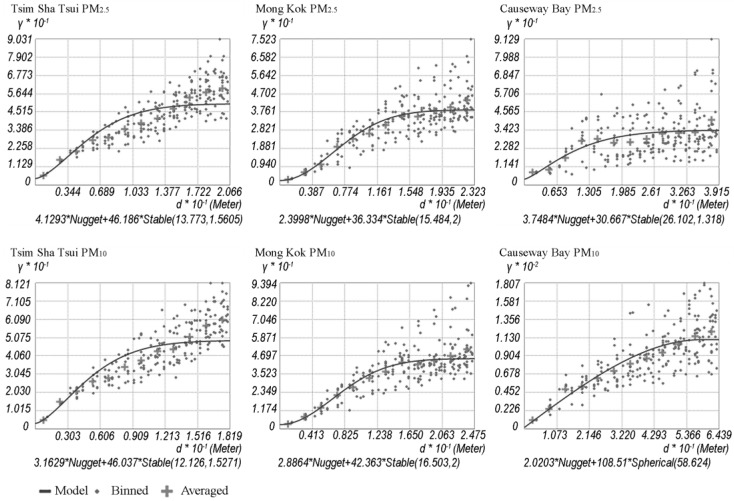
The six resultant semivariogram models established for PM_2.5_ and PM_10_ of the three study areas.

Semivariogram modelling is an essential step for geointerpolation [58]. The bin size selection and the model optimization were determined by taking the advantage of ArcGIS Geostatistical Analyst [59]. This module is able to fit an optimized semivariogram model automatically. Figure 4 shows the six resultant semivariogram models developed for the mapping of the PM_2.5_ and PM_10_ spatial variability in the three districts. They were used as the basis of further interpolation. The geostatistical analysis of semivariogram modeling for the three study areas shows that the “Stable” type is the optimal semivariogram model type of almost all resultant models (except the model of PM_10_ in Causeway Bay which has a “Spherical” model type). The major range of the measured spatial PM_2.5_ and PM_10_ data is range from 12 m (PM_2.5_ in Tsim Sha Tsui) to 58 m (PM_10_ in Causeway Bay). The average level of the major range is approximately 25 m, which is much smaller than the findings in the previous vehicular mobile monitoring study [60]. 

### 3.3. The Comparison of Prediction Performance of the Interpolation Methods

The RMSE and the $R{\;}_{k-fold}^{2}$ of the 13 different types of interpolation methods were compared (grouped by the interpolation algorithms and the weight factors/covariates). Table 3 shows the comparison results of the averaged RMSE and the $R{\;}_{k-fold}^{2}$ of predicted values among the four basic interpolation algorithms. Except for the PM_2.5_ in the Causeway Bay area, the OK method produces the minimum RMSE and also the highest $R{\;}_{k-fold}^{2}$ values for almost all other predicted values of PM_2.5_ and PM_10_. Overall, the OK method shows the best estimation accuracy among all methods in almost all study areas. LPI shows the lowest prediction performance. The performance of the OK, OCK and KIB methods is similar.

Table 4 shows the results and a comparison of the averaged RMSE and the average $R{\;}_{k-fold}^{2}$ of predicted values produced by considering the different weight factors/covariates. The findings by this present study at the microenvironmental scale is different from the conclusion in our previous studies about urban-scale air quality mapping [16,52]. Our previous studies found that the incorporation of urban morphological factors in the geospatial modelling significantly improves the estimation accuracy of the spatial variation of PM concentration at the urban scale. However, it can be observed in this present study that only slight improvements in the interpolation performance were achieved in all three study areas when the fine-scale building geometrical features were considered in the interpolation model (either by using them as weight factors for LPI or as covariates for cokriging). This finding also implies the multiscale properties of the urban outdoor PM exposure in Hong Kong.

### 3.4. The Geospatial Mapping and the Validation

**Figure 5:**
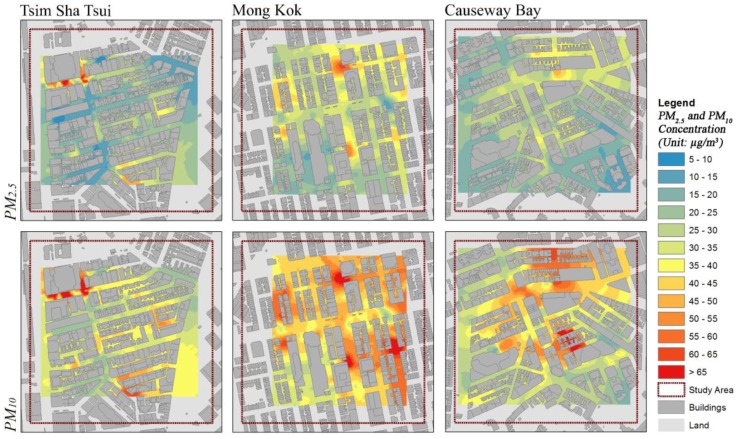
The resultant interpolation mapping of the PM_2.5_ and PM_10_ concentration in all three study areas.

**Figure 6:**
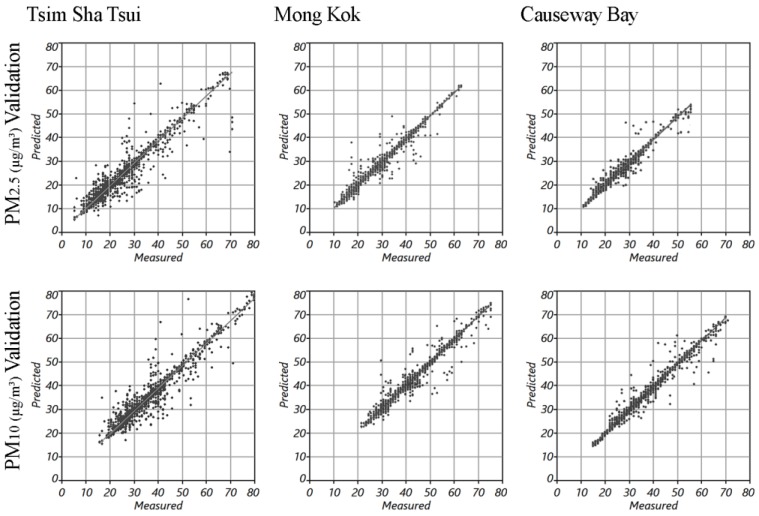
The validation of all resultant interpolation mappings of the PM_2.5_ and PM_10_ concentration in the three districts.

The fine-scale spatial variability of the PM_2.5_ and PM_10_ in the three study areas were mapped using the optimal interpolation methods (with the best prediction performance and the minimum RMSE and the highest $R{\;}_{k-fold}^{2}$ values). The spatial resolution of the resultant prediction surface of each study area was automatically determined by the algorithm embedded in the ArcGIS Geostatistical Analyst based on the measured data. Therefore, the spatial resolution is slightly different for the three study areas (and they are not the integer as well). The resultant spatial resolution of the prediction surfaces range from 1.4 to 1.7 m. Figure 5 illustrates the geospatial interpolation mapping of the PM_2.5_ and PM_10_ concentration in the three study areas. The high PM concentration hotspots can be clearly observed from the mapping results of each of the study areas. The validation results (Figure 6) show that all interpolation mappings of the PM_2.5_ and PM_10_ in the three study areas achieved a satisfying prediction performance.

## 4. Discussion

Studying the microenvironmental scale of air pollution is essential in the assessment of human exposure of Hong Kong residents to air pollution. This present study is a pilot test to depict the fine-scale spatial variability of PM in an extremely compact built environment using geospatial interpolation techniques. A previous local attempt has been made to understand the microenvironmental scale human PM exposure in several districts in Hong Kong [26]. There are also attempts at measuring the spatial variation of ground-level PM by conducting mobile monitoring method [16,61]. However, a major limitation of these prior studies is that the fine-scale spatial variation of pedestrian level PM is not fully depicted. The fixed monitoring and vehicular mobile monitoring cannot effectively detect the PM hotspots caused by the localized emission sources in the study areas. To overcome all the above limitations, we conducted a microenvironmental scale mapping of the spatial variability of PM_2.5_ and PM_10_ using geospatial interpolation mapping based on the measured pedestrian level PM data. It could provide more detailed information in the representation of the pedestrian PM exposure. 

### 4.1. Spatial Variability within Districts—The Necessity of a Multiscale Understanding

According to the results, the average level of the spatial scale of the PM variability (measured as the major range) is approximately 25 m—much smaller than the findings in the previous vehicular mobile monitoring study [61]. This finding confirms that the fine-scale spatial variability of pedestrian level PM can only be effectively monitored by a personal level exposure measuring unit. Typically, the PM_2.5_ concentration at road center should be higher than at the roadside because of the dominance of the on-road vehicular pollutant emission. However, the phenomenon that the PM_2.5_ concentration was higher at the sidewalks but lower at the center of some streets were observed in the resultant mappings of this present study. The PM_2.5_ concentration at is not necessarily lower than the center of the streets. Many of fine-scale PM pollution sources are located on the roadside such as bus stops, parking entrance, cargo areas, and ventilation discharge outlets of restaurants/commercial cooking. They all have a considerably higher emission intensity of PM_2.5_. For example, it has been measured by our previous work that a couple of buses parked at a roadside bus stop could lead to an abnormally high PM_2.5_ concentration [16]. A majority of the large number of restaurants, most of them Chinese [62], are located at the ground-level of a high-rise building podium on the roadside. They stand next to a narrow pedestrian sidewalk, with ventilation discharge outlets along the roadside.

The above findings also imply that a set of multiscale measures must be taken to control the urban outdoor PM exposure level in Hong Kong. To be more specific, urban environmental planning strategies are necessary to enhance the dispersion of PM at the urban scale. Meanwhile, the fine-scale emission sources in the urban downtown area (for example, the densely distributed bus stops [27], restaurants and commercial cooking [28]) also have to be properly inventoried and regulated.

### 4.2. The Difference in Determinants of the Urban Air Pollution Spatial Variability

Continuing the above discussion, the spatial variation of urban air pollution is multiscale [63,64], which means that the dominant factors and determinants of the spatial variability at different spatial scales (i.e., at the street scale and urban scale) are also different. Our previous study confirms that the urban morphological/building geometrical factors significantly determine the spatial distribution of PM_2.5_ and PM_10_ concentration and also the spatial distribution of many other kinds of air pollutants at the urban scale in the compact urban environment of Hong Kong [52]. It is due to the influence of urban surface aerodynamic properties in the urban boundary layer climate and the atmospheric pollutants dispersion. However, the dominant effect of building geometrical features does not appear in the geospatial interpolation mapping of pedestrian level PM at the microenvironmental scale (a much smaller spatial scale of than the urban scale). Only slight improvements in the interpolation performance were achieved in all three study areas when the fine-scale building geometrical features were considered in the interpolation model either by using them as weight factors/covariates. For each study area, several high PM concentration hotspots can be clearly observed from the mapping results (Figure 5). Currently, there is still no well-established inventory/database of fine-scale air pollution emission sources in Hong Kong. In such case, conducting site survey could be a way of investigating the causes of these hotspots. By comparatively analyzing the interpolation mapping results of the PM in each study area and corresponding video records during the measurement campaigns and the information gathered during the site survey, it has been found that the spatial distribution of high PM concentration hotspot locations is highly consistent with the locations of local PM pollution sources (mainly includes busy street/crossroads, bus stops, parking entrance, cargo areas, and those ventilation discharge outlet of restaurants/commercial cooking). The above also supports the argument that the fine-scale emission sources in the urban downtown area must be properly inventoried and regulated to improve the pedestrian level air quality.

### 4.3. Outlook for a Feasible Way of Mapping the Fine-Scale Spatial Variability in Air Pollution Exposure Using Big Data

The geospatial interpolation was adopted to explore the pedestrian-level air pollution concentration in three representative commercial districts of Hong Kong at the microenvironmental scale with the PM data collected by the individual walking-based measurement campaigns. The good mapping accuracy and reasonable validation results of this study (Figure 6) prove that the measured data from the individual measurement unit are competent at providing information for the depiction of fine-scale spatial variability of urban air pollution. Compared with the conventionally fixed measurement at sparsely distributed monitoring locations, using the individual measurement is a more cost-efficient way to provide more detailed spatial information. More importantly, by measuring the spatial variability of air pollution using the individual walking-based measurement, this study shows the high feasibility of creating big data of spatial information on urban air quality based on individual air pollution exposure measurement by regular residents. With the rapid improvement in air quality monitoring technology, air quality sensors are becoming much more portable. Mobile communication devices such as smart phones and tablets with cellular/WIFI signals make it possible to upload real-time air quality monitoring data from the portable air quality sensors for the building up of big data. These big data could be extremely precious and useful for the human exposure assessment and urban environmental management. With the big data on urban air quality with abundant spatial information, the prediction accuracy of the Hong Kong Air Quality Health Index (AQHI) [65] could possibly be promoted to a new level.

### 4.4. Limitations and Future Works

It should be noticed that the geointerpolation is still based on the assumption of a certain function of distance, which largely relies on mathematics and algorithms. A numerical modelling with comprehensive environmental considerations could lead to more robust estimates. However, most of the street canyon studies are using idealized cases for parametric comparison study [46,66]. As discussed, it is challenging to use numerical modelling to depict a real site without a comprehensive inventory of small-scale air pollution emission sources. In such case, although there are inherent uncertainties of the geointerpolation, the personal exposure measurement and the geospatial interpolation of this present study still provide valuable information about the fine-scale spatial variability of the pedestrian level PM_2.5_ concentration. This initial study is a starting point for a comprehensive investigation of small-scale spatial variability of air pollution and evaluation of pedestrian level personal exposure. Further works should and will focus on refining the method, evaluating its uncertainties where possible, understanding its sensitivities to the changes in technical details of the measurement campaign and the input data. Further fine-tuning should be performed to keep improving the robustness of the interpolation results.

## 5. Conclusions

This present study is an initial attempt to investigate the fine-scale spatial variability of the pedestrian level PM in a compact built environment using geospatial analysis methods with real measured individual PM exposure data. Using a self-assembled backpack outdoor environmental measuring unit, we investigated the fine-scale spatial variability of PM in three of the most representative commercial districts of Hong Kong. The geospatial interpolation was then used to analyse the data and to map the microenvironmental PM spatial variation. The results show that the original cokriging with the exponential kernel function provides the best performance in the PM mapping. Using the fine-scale building geometrical features as covariates slightly improves the interpolation performance. The study results also imply that the fine-scale, localized pollution emission sources heavily influence pedestrian exposure to PM. The validation results confirm that the microenvironmental scale mapping of pedestrian-level PM provides better spatial information for exposure assessment and environmental management.

## Figures and Tables

**Table 1:** List of the 13 types of spatial interpolation methods used in this study.

The 13 Types of Methods	Basic Interpolation Algorithm ^1^	Weight Factors ^2^	Covariates ^2^
LPI	LPI	none	n/a
LPI_SVF_	LPI	1	n/a
LPI_FAI_	LPI	2	n/a
LPI_RDA_	LPI	3	n/a
OK	OK	n/a	none
OCK_SVF_	OCK	n/a	1
OCK_FAI_	OCK	n/a	2
OCK_RDA_	OCK	n/a	3
OCK_ALL_	OCK	n/a	1, 2, 3
KIB	KIB	none	n/a
KIB_SVF_	KIB	1	n/a
KIB_FAI_	KIB	2	n/a
KIB_RDA_	KIB	3	n/a

**^1^** The basic interpolation algorithm: Local polynomial interpolation (LPI), Original kriging (OK), Original cokriging (OCK) and Kernel smoothing interpolation with barriers (KIB); **^2^** The weight factors and covariates: **(1)** Sky-view factor within 50-m buffer (SVF_50m_), **(2)** Frontal-area index within 50-m buffer (FAI_50m_), and **(3)** Road-area ratio within 50-m buffer (RDA_50m_).

**Table 2:** The kernel function comparison based on the RMSE/$R{\;}_{k-fold}^{2}$ of the predicted PM concentration value by the LPI method. The method produces the minimum RMSE and the highest $R{\;}_{k-fold}^{2}$ values of the PM_2.5_ and PM_10_ estimation of the three study areas were italicized.

	Study Areas	Tsim Sha Tsui		Mong Kok		Causeway Bay	
Kernel Functions		PM_2.5_ (μg/m^3^)	PM_10_ (μg/m^3^)	PM_2.5_ (μg/m^3^)	PM_10_ (μg/m^3^)	PM_2.5_ (μg/m^3^)	PM_10_ (μg/m^3^)
Exponential		***5.831/0.866***	***6.124/0.863***	***4.577/0.743***	***4.846/0.793***	***2.983/0.729***	***5.372/0.854***
Polynomial5		6.313/0.840	6.645/0.839	4.816/0.711	5.099/0.769	3.166/0.696	5.645/0.844
Gaussian		6.073/0.854	6.387/0.853	4.767/0.719	5.045/0.775	3.169/0.691	5.668/0.837
Epanechnikov		6.825/0.775	7.216/0.771	5.347/0.657	5.643/0.724	3.612/0.560	6.406/0.766
Quartic		6.513/0.815	6.865/0.813	4.976/0.695	5.263/0.755	3.316/0.649	5.895/0.818
Constant		7.071/0.725	7.479/0.723	6.087/0.574	6.383/0.657	4.051/0.436	7.166/0.686

**Table 3:** The Comparison of the RMSE/$R{\;}_{k-fold}^{2}$ of PM concentration levels by the different interpolation algorithms. The method produces the minimum RMSE and the highest $R{\;}_{k-fold}^{2}$ values of the PM_2.5_ and PM_10_ estimation of the three study areas were italicized.

	StudyAreas	Tsim Sha Tsui		Mong Kok		Causeway Bay	
Algorithms		PM_2.5_ (μg/m^3^)	PM_10_ (μg/m^3^)	PM_2.5_ (μg/m^3^)	PM_10_ (μg/m^3^)	PM_2.5_ (μg/m^3^)	PM_10_ (μg/m^3^)
LPI		5.820/0.869	6.202/0.861	4.581/0.736	4.850/0.794	3.051/0.732	5.508/0.848
OK		***4.568/0.917***	***4.764/0.909***	***3.566/0.842***	***3.753/0.867***	2.070/0.883	***3.789/0.924***
OCK		4.647/0.913	4.858/0.905	3.578/0.841	3.771/0.866	2.155/0.875	3.794/0.923
KIB		4.733/0.908	4.943/0.903	3.631/0.835	3.818/0.863	***2.064/0.885***	3.853/0.922

**Table 4:** The comparison of the RMSE/$R{\;}_{k-fold}^{2}$ of PM concentration levels by the consideration either on different weight factors or different covariates. The method produces the minimum RMSE and the highest $R{\;}_{k-fold}^{2}$ values of the PM_2.5_ and PM_10_ estimation of the three study areas were italicized.

	Study Areas	Tsim Sha Tsui		Mong Kok		Causeway Bay	
Covariates/Weight Factors		PM_2.5_ (μg/m^3^)	PM_10_ (μg/m^3^)	PM_2.5_ (μg/m^3^)	PM_10_ (μg/m^3^)	PM_2.5_ (μg/m^3^)	PM_10_ (μg/m^3^)
None		5.043/0.898	5.361/0.890	3.987/0.803	4.205/0.840	2.434/0.828	4.436/0.895
SVF_50m_ (1)		***5.040/0.898***	5.295/0.893	3.922/0.807	4.134/0.843	***2.374/0.838***	***4.350/0.899***
FAI_50m_ (2)		5.061/0.897	***5.287/0.894***	3.907/0.810	4.122/0.844	2.447/0.826	4.394/0.897
RDA_50m_ (3)		5.079/0.895	5.334/0.891	***3.895/0.812***	***4.111/0.844***	2.378/0.837	4.355/0.899

## References

[1] Schwela D., Haq G., Huizenga C., Han W.J., Fabian H. (2012). Urban Air Pollution in Asian Cities: Status, Challenges and Management.

[2] Fenger J., Hertel O., Palmgren F. (1998). Urban Air Pollution-European Aspects.

[3] Mayer H., Haustein C., Matzarakis A. (1999). Urban air pollution caused by motor-traffic. Adv. Air Pollut..

[4] Dockery D.W. (2009). Health effects of particulate air pollution. Ann. Epidemiol..

[5] Pope C.A., Dockery D.W., Schwartz J. (1995). Review of epidemiological evidence of health effects of particulate air pollution. Inhal. Toxicol..

[6] Eliasson I., Offerle B., Grimmond C.S.B., Lindqvist S. (2006). Wind fields and turbulence statistics in an urban street canyon. Atmos. Environ..

[7] Ahmad K., Khare M., Chaudhry K. (2005). Wind tunnel simulation studies on dispersion at urban street canyons and intersections—A review. J. Wind Eng. Ind. Aerodyn..

[8] Hoshiko T., Nakajima F., Prueksasit T., Yamamoto K. (2012). Health risk of exposure to vehicular emissions in wind-stagnant street canyons. Ventilating Cities.

[9] Hills P., Barron W. (1997). Hong Kong: The challenge of sustainability. Land Use Policy.

[10] Fan X., Lam K.-C., Yu Q. (2012). Differential exposure of the urban population to vehicular air pollution in Hong Kong. Sci. Total Environ..

[11] Lee S.C., Cheng Y., Ho K.F., Cao J.J., Louie P.K.K., Chow J.C., Watson J.G. (2006). PM_1.0_ and PM_2.5_ characteristics in the roadside environment of Hong Kong. Aerosol Sci. Technol..

[12] Chan L.Y., Wu H.W.Y. (1993). A study of bus commuter and pedestrian exposure to traffic air pollution in Hong Kong. Environ. Int..

[13] Wong T.W., Tam W.S., Yu T.S., Wong A.H.S. (2002). Associations between daily mortalities from respiratory and cardiovascular diseases and air pollution in Hong Kong, China. Occup. Environ. Med..

[14] Tam W.W.S., Wong T.W., Wong A.H.S. (2015). Association between air pollution and daily mortality and hospital admission due to ischaemic heart diseases in Hong Kong. Atmos. Environ..

[15] Park Y.M., Kwan M.-P. (2017). Individual exposure estimates may be erroneous when spatiotemporal variability of air pollution and human mobility are ignored. Health Place.

[16] Shi Y., Lau K.K.-L., Ng E. (2016). Developing street-level PM_2.5_ and PM_10_ land use regression models in high-density Hong Kong with urban morphological factors. Environ. Sci. Technol..

[17] HKEPD Air Quality Monitoring Network of Hong Kong. http://www.aqhi.gov.hk/en/monitoring-network/air-quality-monitoring-network.html.

[18] Tsang H., Kwok R., Miguel A.H. (2008). Pedestrian exposure to ultrafine particles in Hong Kong under heavy traffic conditions. Aerosol Air Qual. Res..

[19] Chan L.Y., Kwok W.S. (2001). Roadside suspended particulates at heavily trafficked urban sites of Hong Kong—Seasonal variation and dependence on meteorological conditions. Atmos. Environ..

[20] So K.L., Guo H., Li Y.S. (2007). Long-term variation of PM_2.5_ levels and composition at rural, urban, and roadside sites in Hong Kong: Increasing impact of regional air pollution. Atmos. Environ..

[21] Lam G., Leung D., Niewiadomski M., Pang S., Lee A., Louie P. (1998). Street-level concentrations of nitrogen dioxide and suspended particulate matter in Hong Kong. Atmos. Environ..

[22] Juhos I., Makra L., Tóth B. (2008). Forecasting of traffic origin no and no2 concentrations by support vector machines and neural networks using principal component analysis. Simul. Model. Practice Theory.

[23] Lu W.Z., Wang W.J., Wang X.K., Xu Z.B., Leung A.Y.T. (2003). Using improved neural network model to analyze rsp, nox and no2 levels in urban air in mong kok, Hong Kong. Environ. Monit. Assess..

[24] Kwan M.-P. (2012). The uncertain geographic context problem. Ann. Assoc. Am. Geogr..

[25] Yoo E., Rudra C., Glasgow M., Mu L. (2015). Geospatial estimation of individual exposure to air pollutants: Moving from static monitoring to activity-based dynamic exposure assessment. Ann. Assoc. Am. Geogr..

[26] Chan L.Y., Kwok W.S., Lee S.C., Chan C.Y. (2001). Spatial variation of mass concentration of roadside suspended particulate matter in metropolitan Hong Kong. Atmos. Environ..

[27] Chan L.Y., Chan C.Y., Qin Y. (1999). The effect of commuting microenvironment on commuter exposures to vehicular emission in Hong Kong. Atmos. Environ..

[28] Lee S.C., Li W.-M., Yin Chan L. (2001). Indoor air quality at restaurants with different styles of cooking in metropolitan Hong Kong. Sci. Total Environ..

[29] Marshall S. (2004). Streets and Patterns.

[30] Grimmond C.S.B. (1998). Aerodynamic roughness of urban areas derived from wind observations. Bound.-Layer Meteorol..

[31] Kastner-Klein P., Rotach M. (2004). Mean flow and turbulence characteristics in an urban roughness sublayer. Bound.-Layer Meteorol..

[32] Bottema M. (1997). Urban roughness modelling in relation to pollutant dispersion. Atmos. Environ..

[33] HKEPD Air Quality Monitoring Stations info. http://www.aqhi.gov.hk/en/monitoring-network/air-quality-monitoring-stations9c57.html?stationid=81.

[34] Ramachandran G., Adgate J.L., Pratt G.C., Sexton K. (2003). Characterizing indoor and outdoor 15 minute average PM_2.5_ concentrations in urban neighborhoods. Aerosol Sci. Technol..

[35] Ng E. (2009). Policies and technical guidelines for urban planning of high-density cities—Air ventilation assessment (ava) of Hong Kong. Build Environ..

[36] Yuan Z., Lau A.K.H., Zhang H., Yu J.Z., Louie P.K.K., Fung J.C.H. (2006). Identification and spatiotemporal variations of dominant PM_10_ sources over Hong Kong. Atmos. Environ..

[37] Lau A., Lo A., Gray J., Yuan Z., Loh C. (2007). Relative Significance of Local vs. Regional Sources: Hong Kong’s Air Pollution.

[38] Cheng Y., Ho K.F., Lee S.C., Law S.W. (2006). Seasonal and diurnal variations of pm_1.0_, PM_2.5_ and PM_10_ in the roadside environment of Hong Kong. China Part..

[39] Ng E., Cheng V., Gadi A., Mu J., Lee M., Gadi A. (2007). Defining standard skies for Hong Kong. Build Environ..

[40] Kukkonen J., Pohjola M., Sokhi R.S., Luhana L., Kitwiroon N., Fragkou L., Rantamäki M., Berge E., Ødegaard V., Håvard Slørdal L. (2005). Analysis and evaluation of selected local-scale PM_10_ air pollution episodes in four european cities: Helsinki, London, Milan and Oslo. Atmos. Environ..

[41] Hong Kong Observatory (HKO) (2015). Monthly Meteorological Normals for Hong Kong, 1981–2010.

[42] Penwarden A.D., Wise A.F.E. (1975). Wind Environment around Buildings.

[43] Li J., Heap A.D. (2008). A Review of Spatial Interpolation Methods for Environmental Scientists.

[44] Oke T.R. (2004). Initial Guidance to Obtain Representative Meteorological Observations at Urban Sites.

[45] Van Donkelaar A., Martin R.V., Park R.J. (2006). Estimating ground-level PM_2.5_ using aerosol optical depth determined from satellite remote sensing. J. Geophys. Res. Atmos..

[46] Vardoulakis S., Fisher B.E.A., Pericleous K., Gonzalez-Flesca N. (2003). Modelling air quality in street canyons: A review. Atmos. Environ..

[47] Briggs D.J., Collins S., Elliott P., Fischer P., Kingham S., Lebret E., Pryl K., van Reeuwijk H., Smallbone K., Van Der Veen A. (1997). Mapping urban air pollution using gis: A regression-based approach. Int. J. Geogr. Inf. Sci..

[48] Man Sing W., Nichol J., Kwon-Ho L., Zhanqing L. Retrieval of Aerosol Optical Thickness using Modis 500 × 500 m^2^, a Study in Hong Kong and Pearl River Delta Region. Earth Observation and Remote Sensing Applications.

[49] Chengcai L., Lau A.K.H., Jietai M., Chu D.A. (2005). Retrieval, validation, and application of the 1-km aerosol optical depth from modis measurements over Hong Kong. IEEE Trans. Geosci. Remote Sens..

[50] Gousseau P., Blocken B., Stathopoulos T., van Heijst G.J.F. (2015). Near-field pollutant dispersion in an actual urban area: Analysis of the mass transport mechanism by high-resolution large eddy simulations. Comput. Fluids.

[51] Shi Y., Ren C., Zheng Y., Ng E. (2016). Mapping the urban microclimatic spatial distribution in a sub-tropical high-density urban environment. Archit. Sci. Rev..

[52] Shi Y., Lau K.K.-L., Ng E. (2017). Incorporating wind availability into land use regression modelling of air quality in mountainous high-density urban environment. Environ. Res..

[53] Webster R., Oliver M. (2001). Geostatistics for Environmental Scientists (Statistics in Practice).

[54] Hengl T. (2007). A practical guide to geostatistical mapping of environmental variables. JRC Scientific and Technical Reports.

[55] Lam N.S.N. (1983). Spatial interpolation methods: A review. Amer. Cartographer..

[56] Johnston K. (2004). Arcgis 9: Using Arcgis Geostatistical Analyst.

[57] Refaeilzadeh P., Tang L., Liu H., Liu L., ÖZsu M.T. (2009). Cross-validation. Encyclopedia of Database Systems.

[58] Olea R.A. (2006). A six-step practical approach to semivariogram modeling. Stoch. Environ. Res. Risk Assess..

[59] ESRI Choosing a Lag Size. http://desktop.arcgis.com/en/desktop/latest/guide-books/extensions/geostatistical-analyst/choosing-a-lag-size.htm.

[60] Lau K.K.-L., Ren C., Shi Y., Zheng V., Yim S., Lai D. Determining the optimal size of local climate zones for spatial mapping in high-density cities. Proceedings of the 9th International Conference on Urban Climate jointly with 12th Symposium on the Urban Environment.

[61] Lightowlers C., Nelson T., Setton E., Keller C.P. (2008). Determining the spatial scale for analysing mobile measurements of air pollution. Atmos. Environ..

[62] He L.-Y., Hu M., Huang X.-F., Yu B.-D., Zhang Y.-H., Liu D.-Q. (2004). Measurement of emissions of fine particulate organic matter from chinese cooking. Atmos. Environ..

[63] Oke T.R. (1997). Urban Environments.

[64] Zannetti P. (1990). Air Pollution Modeling: Theories, Computational Methods, and Available Software.

[65] Wong T.W., Tam W.W.S., Yu I.T.S., Lau A.K.H., Pang S.W., Wong A.H.S. (2013). Developing a risk-based air quality health index. Atmos. Environ..

[66] Li X.-X., Liu C.-H., Leung D.Y.C., Lam K.M. (2006). Recent progress in cfd modelling of wind field and pollutant transport in street canyons. Atmos. Environ..

